# Supplementary material for: OXPHOS remodeling in high-grade prostate cancer involves mtDNA mutations and increased succinate oxidation
Source: Nat Commun. 2020 Mar 20;11:1487. doi: 10.1038/s41467-020-15237-5 (PMC7083862; doi:10.1038/s41467-020-15237-5)
Supplement: Supplementary file 1 — Supplementary Information [file 41467_2020_15237_MOESM1_ESM.pdf]

Supplementary Information

OXPHOS remodeling in high-grade prostate cancer involves mtDNA mutations and increased succinate oxidation

Schöpf et al.

Supplementary Tables

Supplementary Table 1 Respiratory states and flux control ratios.

The substrate-uncoupler-inhibitor titration protocol (SUIT-028, http://www.mitofit.org/index.php/SUIT-028) provides a sequential analysis of mitochondrial respiratory capacity in electron transfer-pathway states and specific respiratory coupling control states[1, 2, 3]. Substrates: [1] Glutamate, G, and Malate, M, inducing the LEAK state (GM_L); [2] ADP, inducing the OXPHOS state (GM_P); subsequently oxidative stress by H_2O_2 for 15 min followed by H_2O_2 inactivation by catalase; [3] Pyruvate, P (PGM_P or N_P); [4] Succinate, S (NS_P). Uncoupler: [5] FCCP, U, inducing the ET state (NS_E). Inhibitors inducing the state of residual oxygen consumption, ROX (Rox): [6] Rotenone, Rot (S_E); [7] CI inhibitor Malonate, Mna; CII inhibitor Antimycin A, Ama. Single CIV capacity measurement: [8] Ascorbate, As and N,N,N',N'-Tetramethyl-p-phenylenediamine dihydrochloride, TMPD for activation of CIV; [9] azide, Azd for inhibition of CIV and chemical background O_2 consumption.

Step: Metabolic control variable	Abbreviation concentration	Pathway (Q-junction)	Respiratory state	Ref. state SUIT protocol	Flux control ratio, *FCR*	Significance
**1: glutamate & malate**	G 10 mM &<br>M 2 mM	N (CI)	GM_L	NS_E	GM_L/NS_E	N-pathway (GM), LEAK respiration
**2: ADP**<br>**H_2O_2, oxid. stress**	D 2.5 mM<br>0.5 mM (15 min)	N (CI)	$GM_{P,pre}$<br>GM_P	NS_E<br>NS_E	GM_L/NS_E<br>$GM_{P,pre}/NS_E$	N-pathway (GM), OXPHOS capacity<br>GM_P inhibited by oxidative stress
**3: pyruvate**	P 5 mM	N (CI)	$N(PGM)_P$; N_P	NS_E	GM_P/NS_E	N-pathway (PGM), OXPHOS capacity
**4: succinate**	S 10 mM	NS (CI&II)	NS_P	NS_E	N_P/NS_E	NS-pathway (PGMS), OXPHOS capacity
**5: uncoupler (FCCP)**	U 0.5 µM steps	NS (CI&II)	NS_E	NS_E	NS_P/NS_E	NS-pathway (PGMS), ET capacity
**6: rotenone**	Rot 0.5 µM	S (CII)	S_E	NS_E	S_E/NS_E	S-pathway (S), inhibition CI; ET capacity
**7: malonate & antimycin A**	Mna 5 mM &<br>Ama 2.5 µM		ROX	NS_E+*Rox*	*Rox*/(NS_{E+Rox})	inhibition CII<br>inhibition CIII; residual O_2 consumption
**8: ascorbate & TMPD**	As 2 mM &<br>Tm 0.5 mM		CIV	NS_E	CIV/NS_E	single step CIV activity
**9: azide**	Azd 100 mM		chem. background			inhibition CIV; autoxidation of As&Tm

Supplementary Table 2 Effect of metabolic control variables on respiratory capacities.

Step: Metabolic control variable	Background state	Ref. state Single step	Flux control ratio, *FCR*	Difference of flux [pmol s^{-1} mg^{-1}]	Significance
**1: glutamate & malate**	ROX	GM_L			GM-pathway, LEAK respiration
**2: ADP**<br>**H_2O_2, oxid. stress**	GM_L<br>$GM_{P,pre}$	$GM_{P,pre}$<br>GM_P	$GM_L/GM_{P,pre}$<br>$GM_{P,pre}/GM_P$	$GM_{P,pre}-GM_L$<br>$GM_P-GM_{P,pre}$	OXPHOS efficiency<br>inhibition by oxidative stress
**3: pyruvate**	GM_P	N_P	GM_P/N_P	N_P-GM_P	stimulation by P in CI-convergent N-pathways
**4: succinate**	N_P	NS_P	N_P/NS_P	NS_P-N_P	stimulation by S in CI&II-convergent NS-pathways
**5: uncoupler**	NS_P	NS_E	NS_P/NS_E	NS_E-NS_P	*E-P* excess capacity
**6: rotenone (inhibition CI)**	S_E	NS_E	S_E/NS_E	NS_E-S_E	stimulation by PGM in CI&II-convergent NS-pathways

Supplementary Table 3 Logistic regression analysis.

a Analysis included all mtDNA heteroplasmies (non-coding, synonymous and non-synonymous). Age [a], free serum PSA ratio [%] and tumor stage (pT) were independent variables. Variables that did not show a correlation with the presence of mtDNA heteroplasmies in univariate analysis were not included. **b** Analysis on non-synonymous heteroplasmies in PCa samples only. High-grade stage (Gleason score>7), age [a] and free serum PSA ratios [%] were independent variables. Variables that did not show a correlation with the presence of non-synonymous mtDNA heteroplasmies in the malignant tissue in univariate analysis were not included. Variable coefficients, standard errors (S.E.), odds ratios (O.R.) including their 95% confidence intervals (Ci) and *p*-values (*p*) are listed. The likelihood-ratio test was used to test for significant correlation. Spearman's rank correlation coefficients were calculated to evaluate the presence of significant correlations among non-parametric variables and to test for multicollinearity among single independent variables used in the logistic regression model.

Variable	Coefficient	S.E.	*p*	O.R. (95% Ci)
		**a All heteroplasmies**		
age at diagnosis [a]	0.089	0.043	0.040	1.093 (1.004 - 1.189)
free PSA [%]	-0.118	0.060	0.048	0.889 (0.790 - 0.999)
pT stage	-0.916	0.538	0.088	0.400 0.139 - 1.147)
		**b Non-synonymous heteroplasmies in PCa tissue only**		
Gleason score >7	2.170	0.958	0.022	8.759 (1.340 – 57.26)
age at diagnosis [a]	-0.084	0.046	0.068	0.920 (0.841 - 1.006)
free PSA [%]	-0.133	0.067	0.045	0.875 (0.768 – 0.997)

Supplementary Table 4 Tissue samples for RNA Seq

Sixteen tumor cases were chosen for mRNA expression analyses based on the relative contribution of GM-pathway OXPHOS capacity (GM_P) to PGMS-OXPHOS capacity. In addition, Gleason-scores were considered. Low GM-pathway capacity, representing a severe respiratory phenotype, was defined as ≤0.27 while high GM-pathway capacity, representing a mild respiratory phenotype, was defined as >0.27, respectively. In the severe phenotype tumors five out of eight samples harbored non-synonymous mtDNA mutations in genes encoding for mt-CI subunits, while only one sample in the mild phenotype subgroup showed a potentially deleterious mutation (in *MT-ND3*). Mt-position: location of HP according to the rCRS; HP: type of nucleotide substitution; HP level [%]: Level of detected nucleotide substitution compared to the rCRS; Gene ID: mt-gene affected by detected HP; AA subst: type of amino acid substitution caused by the mutation.

Sample ID	Gleason score	Relative GM_P-pathway capacity	Non-synonymous mtDNA CA mutation [mtDNA position]	HP level [%]	Gene ID	AA subst
**PK 28**	7	high (0.30)	9460	44.1	*MT-CO3*	L85P
**PK 31**	7	high (0.57)				
**PK 34**	7	high (0.37)	10326	3.8	*MT-ND3*	S90P
**PK 41**	7	high (0.29)				
**PK 44**	7	low (0.27)	5359	30.2	*MT-ND2*	I297T
			13916	2.5	*MT-ND5*	G527E
**PK 46**	9	high (0.30)				
**PK 47**	6	low (0.13)				
**PK 50**	9	low (0.22)	7986	7.7	*MT-CO2*	R134Q
**PK 54**	9	low (0.12)	3850	2.2	*MT-ND1*	A182T
			4896	17.4	*MT-ND2*	Y143H
			13288	2.6	*MT-ND5*	G318S
**PK 60**	9	low (0.14)				
**PK 62**	7	low (0.17)	10731	5.5	*MT-ND4L*	D88N
**PK 65**	7	high (0.31)	7589	2.1	*MT-CO2*	A2T
			15719	8.7	*MT-CYB*	Y325H
**PK 72**	7	high (0.34)				
**PK 80**	7	low (0.27)	9010	54.0	*MT-ATP6*	A162T
			13886	5	*MT-ND5*	L517P
**PK 82**	10	low (0.12)	10353	60.2	*MT-ND3*	A99T
**PK 90**	6	high (0.28)				

Supplementary Table 5 Progression-free survival and severe mt-respiratory phenotype. Univariate and multivariate Cox Hazards analysis of prostate cancer mRNA expression profile cohorts stratified by the severe phenotype gene expression signature (Table 2). Gene expression and metadata of PCa cohorts were downloaded from TCGA Data Portal [https://cancergenome.nih.gov/cancersselected/prostatecancer] and Gene Expression Omnibus [https://www.ncbi.nlm.nih.gov/geo/]. Univariate and multivariate Cox regressions were calculated based on the metagene expression signature associated with the severe respiratory phenotype, using an optimized cut-off value yielding the lowest *p*-value (stratified log-rank test).

Progression-free survival and severe mt-respiratory phenotype.

**Cohort** Survival Information	**Variables**	**Univariate Cox model**		**Multivariate Cox model**	
		**HR (95% Ci)**	***p***	**HR (95% Ci)**	***p***
GSE16560 (*N*=281) Overall survival	age			1.05 (1.03-1.08)	<0.001
	Gleason score			1.81 (1.60-2.06)	<0.001
	severe phenotype metagenes	0.61 (0.44-0.84)	0.002	0.92 (0.81-1.03)	0.139
GSE40272 (*N*=84) Disease-free survical	PSA			1.03 (0.99-1.08)	0.1574
	T staging			4.18 (1.17-14.92)	0.028
	N staging			7.51 (1.78-31.79)	0.006
	severe phenotype metagenes	0.48 (0.19-1.23)	0.120	1.23 (0.58-2.60)	0.591
GSE70768 (*N*=111) Biochemical recur-rence-free survival	age			0.80 (0.95-1.12)	0.412
	PSA			1.03 (0.76-1.04)	0.156
	T staging			0.89 (0.58-8.17)	0.248
	Gleason score			2.18 (1.85-8.17)	0.002
	severe phenotype metagenes	0.31 (0.1-0.94)	0.029	4.98 (0.29-2.19)	0.659
GSE70769 (*N*=94) Biochemical recur-rence-free survival	Gleason score			2.11 (1.50-2.97)	<0.001
	PSA			1.01 (1.50-2.97)	0.511
	T staging			1.47 (0.97-1.06)	0.100
	severe phenotype metagenes	0.29 (0.12-0.57)	<0.001	0.53 (0.29-0.96)	0.037
PRAD-TCGA (*N*=497) Disease-free survival	PSA			1.02 (0.98-1.05)	0.338
	T staging			1.72 (1.03-2.86)	0.037
	N staging			0.83 (0.49-1.43)	0.508
	Gleason score			2.03 (1.56-2.65)	<0.001
	severe phenotype metagenes	0.53 (0.32-0.89)	0.0014	0.61 (0.44-0.84)	0.034

Supplementary Table 6 Recent studies on mtDNA mutations in PCa tissue using NGS

	Lindberg et al.[4]	Ju et al.[5]	McCrow et al.[6]	Kalsbeek et al.[7]	Hopkins et al.[8]	Present study
Method	WES	WGS	mWGS	mWGS	WGS	**mWGS**
Nr. of patients	55	80	61	115	384	**50**
Average depth	~300	~8,000	~3,000	>2,000	>10,000	**>10,000**
HP/mtSNV treshold	N.R.	3%	∆HF>0.02	∆HF>0.1	∆HF>0.2	**2%**
Control tissue	-	-	Blood	Blood	Blood	**Prostate**
HP/mtSNV rate	1.0	2.1	1.9	0.7	0.8‡	**1.6**

‡mtSNV rate is given for all variants irrespective of tissue type

Abbreviations: WES, whole exome sequencing; WGS, whole genome sequencing, mWGS, mtDNA whole genome sequencing; N.R., not reported; ∆HF, HP frequency difference tumor/control tissue.

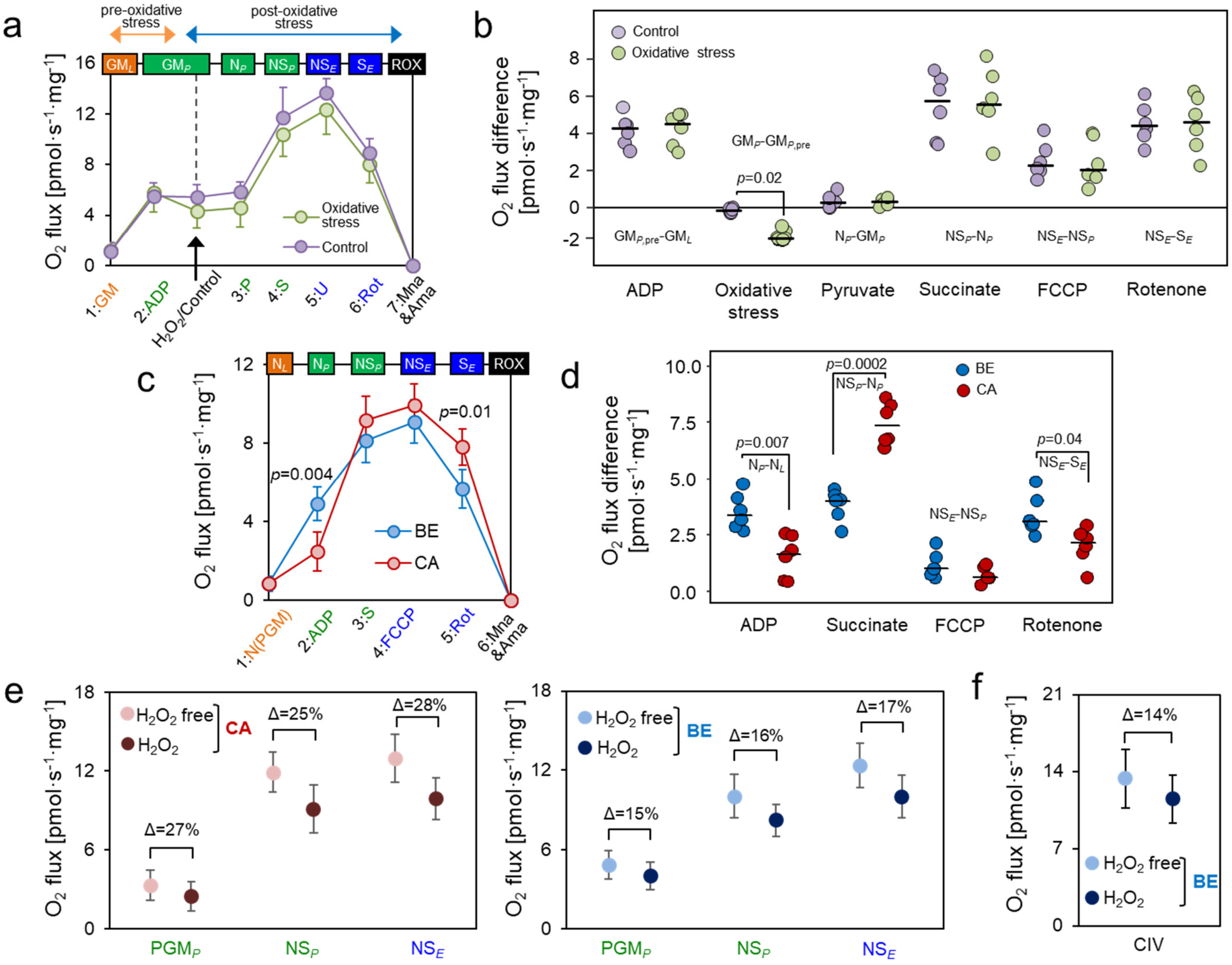

Supplementary Figure1 Effect of oxidative stress on respiratory capacity. a Oxidative stress test by 15 min exposure to 500 µM H_2O_2 or H_2O as control. O_2 flux in split BE tissue samples (*N*=6). Data are presented as mean values ± SD. The arrow indicates the titration of H_2O_2 or H_2O into the O2k chamber. **b** Effects of substrates glutamate&malate, ADP, pyruvate, succinate, H_2O_2 or H_2O, uncoupler FCCP, and CI inhibitor rotenone on O_2 flux in split tissue samples subjected to a SUIT protocol with (green) or without (pink) oxidative stress treatment. Data are presented as mean values and single data points. Differences in mean values were tested for significance using Wilcoxon signed-rank test. **c-d** HRR analysis in paired BE/CA tissue samples applying a SUIT protocol without oxidative stress treatment. **c** O_2 flux in the indicated coupling/pathway control states in benign (blue, *N=6*) and malignant (red, *N=6*) tissue samples. Data are presented as mean values ± SD. **d** Effect of substrates, uncoupler and CI-inhibitor rotenone on O_2 flux in the benign (blue, *N=6*) and malignant (red, *N=6*) tissue samples. Data are presented as mean values and single data points. Differences in mean values were tested for significance using Wilcoxon signed-rank test in c-d. **e** Comparison of respiratory capacities in the indicated coupling/pathway control states in paired benign (blue, *N*=6) and tumor (red, *N*=6) samples analyzed without imposed oxidative stress (light colors, *N*=6) compared to paired samples analyzed with inclusion of the oxidative stress step (dark colors, *N*=50). **f** Effect of oxidative stress by H_2O_2 treatment on CIV activity. O_2 flux was measured in split benign samples after treatment with 500 µM H_2O_2 to mimic oxidative stress (dark blue, *N=6*) or H_2O (light blue, *N=6*) as control. Values in e-f represent mean ± SD. See legend to Figure 2 and Supplementary Tables 1-2 for description of the respiratory states and steps.

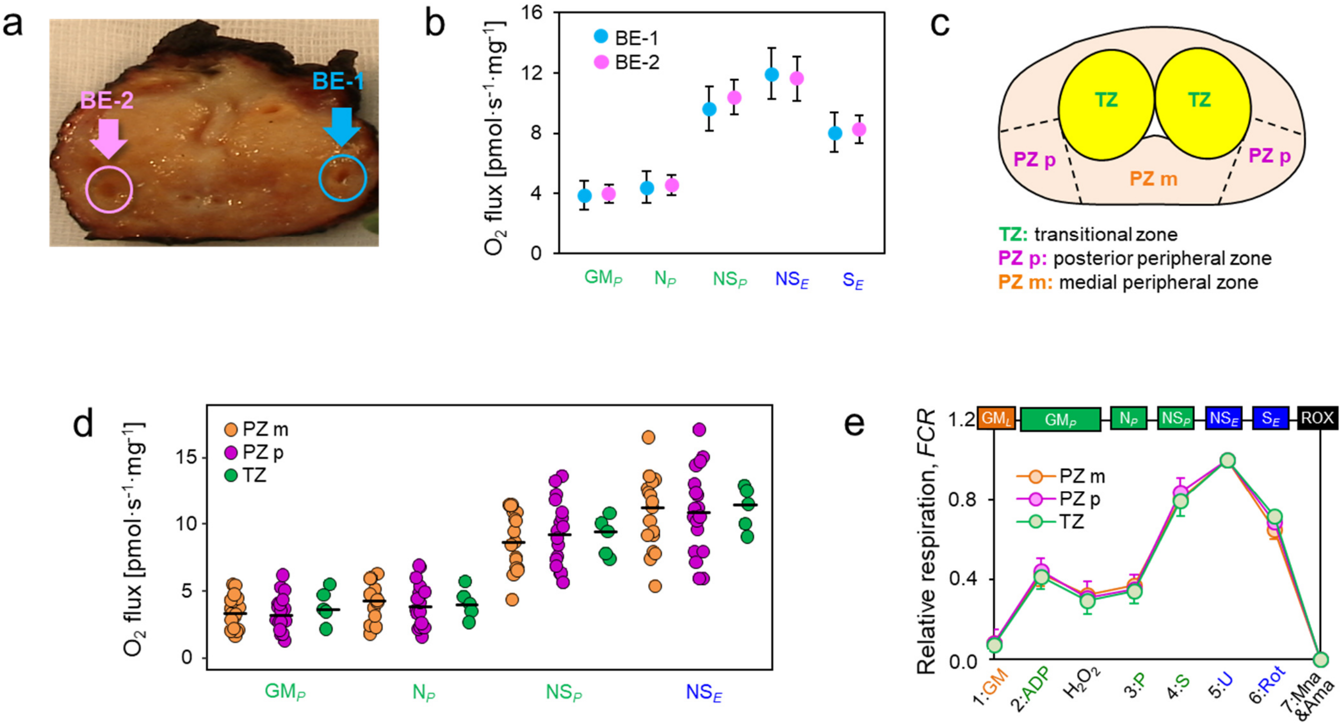

Supplementary Figure 2 OXPHOS capacities in paired benign tissue from different locations. a Paired benign tissue samples (BE-1 and BE-2) from 20 radical prostatectomy specimens were extracted distant from each other, normally from the contralateral site, via punch needle biopsy by a certified uropathologist. Benign status was confirmed by histopathology analysis as shown in Figure 1b. **b** Comparison of OXPHOS- and ET-capacities of indicated states in paired BE/BE (BE1, N=20; BE2, N=20) tissue samples. The same SUIT-028 protocol as for BE/CA paired sample analysis was applied. Data are presented as mean values ± SD. **c** Schematic representation of a prostate cross-section and the zonal architecture. Tissue biopsies for the pairwise comparison of BE-1 and BE-2 samples were obtained from the transitional zone (TZ) or medial or posterior peripheral zone (PZ m and PZ p, respectively). **d** Comparison of OXPHOS- and ET-capacities in benign samples (BE-1 and BE-2) extracted from the PZ m (orange; N=17), the PZ p (magenta; N=18) or the TZ (green; N=5). Mean values and individual data points are presented. **e** Normalized respiratory capacities in benign samples (BE-1 and BE-2) extracted from the PZ m (orange; N=17), the PZ p (magenta; N=18) or the TZ (green; N=5). Values represent mean ± SD. See legend to Figure 2 and Supplementary Tables 1-2 for description of the respiratory states and steps.

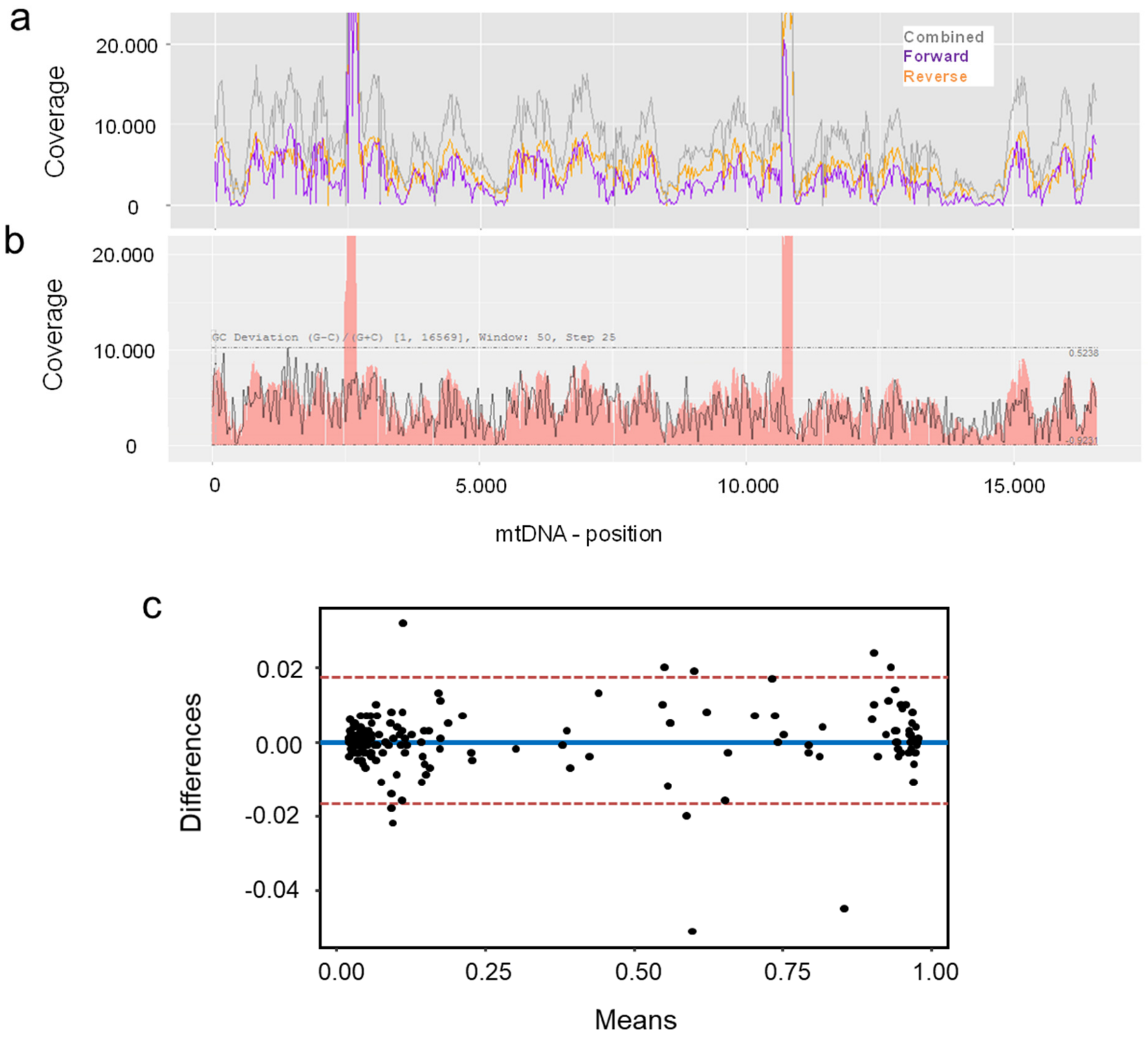

Supplementary Fig. 3 mtDNA sequencing coverage. Mean forward and reverse mtDNA nucleotide coverage, mtDNA coverage correlated to GC content and Bland-Altmann plot of all variants as detected by two independent sequencing runs. **a** Typical coverage plot for one representative sample showing of forward (purple), reverse (orange) and the combined coverage (grey), respectively, along the mtDNA of an Ion Proton sequencing run of 40 barcoded samples. The two high coverage peaks represent the overlapping areas of the two long-range PCR amplicons covering the entire mt-genome. **b** Influence of GC content. The mean nucleotide coverage, here exemplarily of the reverse reads (red area) is highly concordant with the GC content (grey line) of the DNA sequence. This pattern was seen for all samples and all sequencing runs, irrespective of library concentration used for ion sphere particle generation, chip loading density or nucleotide coverage. **c** Bland-Altmann plot of the concordance between the two runs. Values on the *x*-axis represent the mean heteroplasmy (HP) level while values on the *y*-axis represent the differences of the HP levels for each variant between the two runs.

a

Sample	Locus	Variant	RNA alteration	HP level	MitoTIP prediction
PK_30	*MT-TP*	G15995A	U	15%	likely pathogenic
PK_62	*MT-TW*	A5539G	C	12%	likely benign
PK_98	*MT-TF*	T605C	G	18%	Not reported
PK_99	*MT-TQ*	C4380T	A	4%	likely benign

b

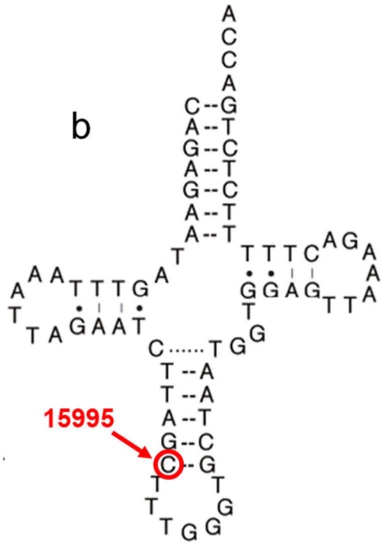

Supplementary Figure 4 MT-tRNA mutations in benign and malignant tissue samples. a List of samples harboring mtDNA encoded tRNA mutations. Listed are the location (*MT-TP*= $tRNA^{Pro}$, MT-TW= $tRNA^{Trp}$, *MT-TF*= $tRNA^{Phe}$, *MT-TQ*= $tRNA^{Glx}$), the variant, the alteration of RNA sequence caused by the variant, the HP level of the variant and the predicted pathogenic effect as determined by MitoTIP (https://www.mitomap.org/cgi-bin/mitotip). **b** Cloverleaf structure of mtDNA encoded $tRNA^{Pro}$. The red circle indicates the location on the anticodon stem affected by the G>A substitution at position 15995. This mutation most likely leads to a destabilization in RNA folding since it is the last base pair required for the stabilization of the anticodon loop.

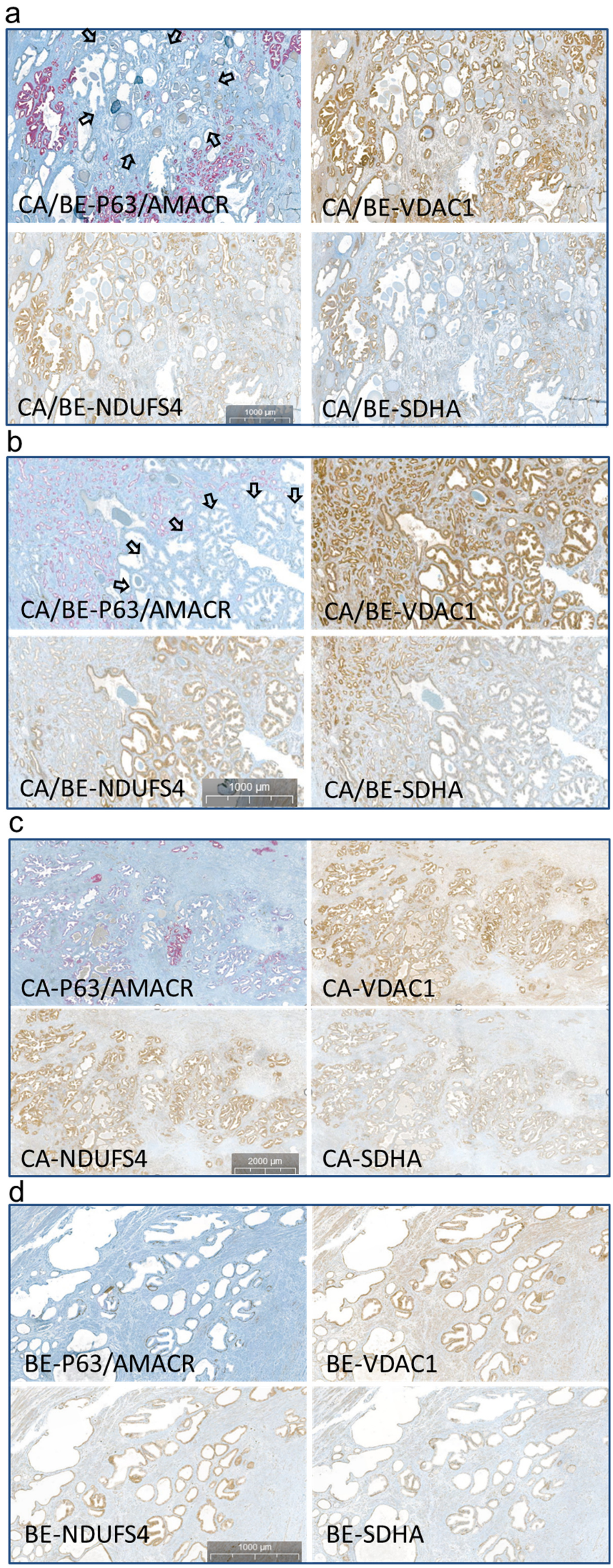

Supplementary Figure 5 IHC expression pattern of mitochondrial markers.

a-d Representative images of IHC immunoreactivity patterns of 3 representative cases of 24 cases in total. Consecutive sections were immunostained with antibodies directed against p63/AMACR (double staining brown and read) to identify tumor and benign regions. A benign region is indicated by arrows in **a** and in **b**, For the third case CA (**c**) and BE (**d**) images are presented. Tumor areas can be recognized by red AMACR staining in the P63/AMACR double stain images. Anti-VDAC1 recognizing porin is a marker for mitochondrial mass, anti NDUFS4 a marker for mitochondrial Complex I and anti-SDHA for mitochondrial Complex II. Altogether, 24 cases were immunostained for evaluation of marker expression. Scale bars indicate 1000 µm (a, b and d) and 2000µm (d), respectively.

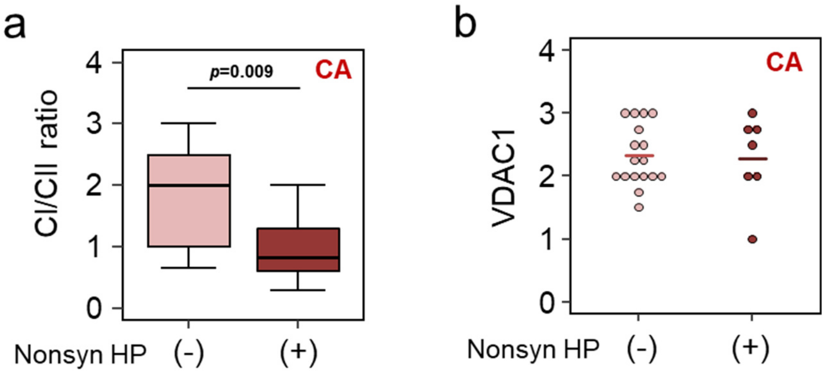

Supplementary Figure 6 IHC Expression pattern of mitochondrial markers. a CI/CII marker immunoreactivity ratio in tumors harboring (+; N=7) or not harboring (-; N=17) mt-CI gene HPs. Data are presented as box blots indicating median, 25th–75th percentile (box) and minimum/maximum (whiskers). Differences in mean values were tested for significance using Wilcoxon rank-sum test. **b** mt-mass marker VDAC1 immunoreactivity in these two tumor cohorts.

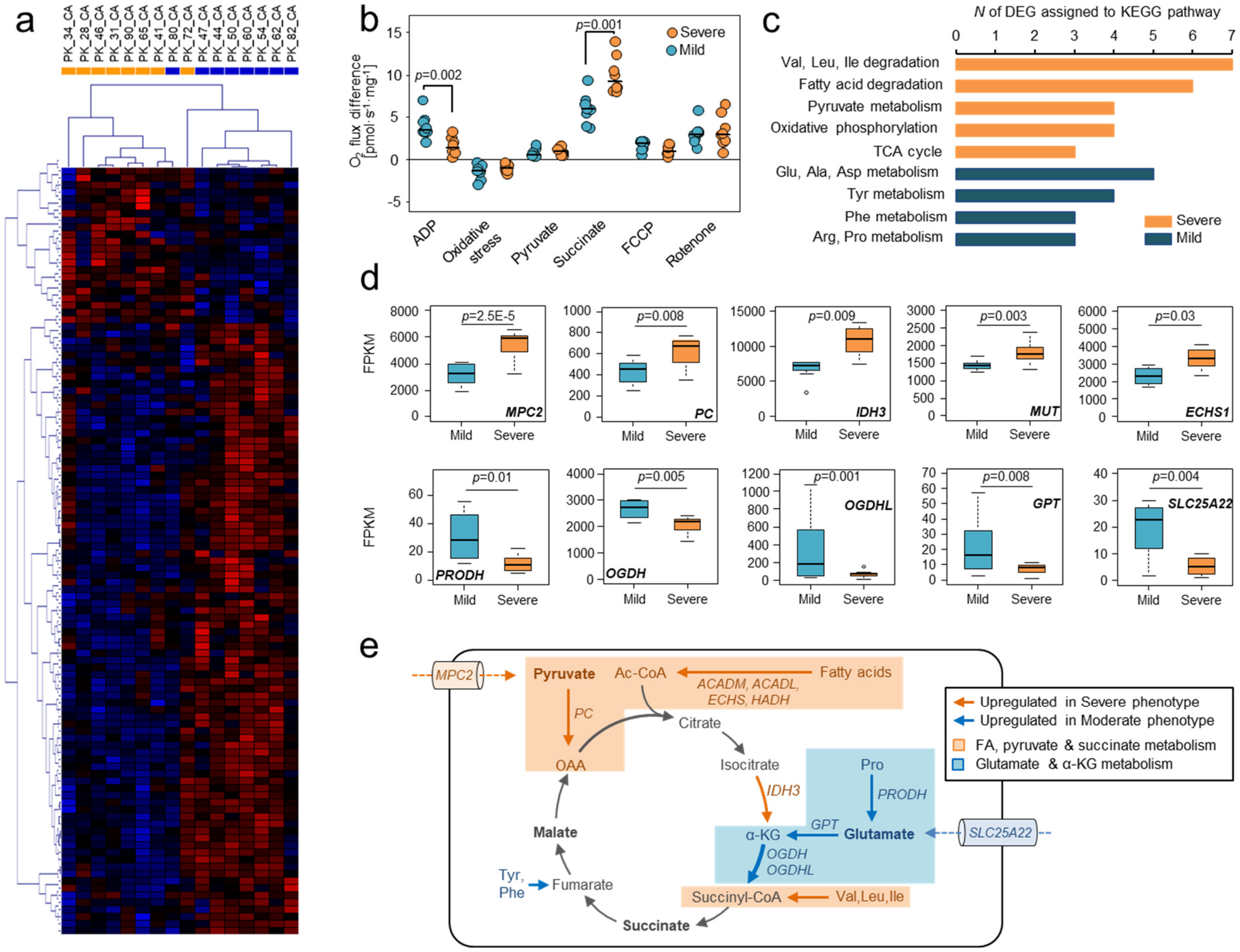

Supplementary Figure 7 Gene expression in severe and mild respiratory phenotype tumors. a Heatmap and hierarchical clustering of all significantly differentially expressed mitochondria-related genes (MitoCarta 2.0 Gene Catalog, *FDR*<0.1, Benjamini-Hochberg procedure) in the severe (orange, *N*=8) or mild (blue, *N*=8) respiratory phenotype tumor samples. **b** Effects of substrates, oxidative stress, uncoupler, and CI inhibitor on O_2 flux in tissue samples used for transcriptome profiling by RNA-seq. Tumor samples were grouped according to their respiratory phenotype in a mild (blue, *N*=8) and severe phenotype (orange, *N*=8) group, respectively (Supplementary Table 4). Mean values and individual data points are presented. Differences in mean values were tested for significance using Wilcoxon signed-rank test. **c** Enriched metabolic KEGG pathways upregulated (orange) or downregulated (blue) in cancer tissue based on the InnateDB pathway over-representation analysis of all differentially expressed mt-related genes. **d** Boxplot representing Fragments Per Kilobase Million (FPKM) values of significantly differently expressed metabolic key-enzymes in tumor samples exhibiting a severe (orange, *N*=8) or mild (light blue, *N*=8) respiratory phenotype (*MPC2*: mitochondrial pyruvate carrier 2; *PC*: pyruvate carboxylase; *IDH3*: isocitrate dehydrogenase 3 subunit gamma; *MUT*: methylmalonyl-CoA mutase; *ECHS1*: enoyl-CoA hydratase, short chain 1; *PRODH*: proline dehydrogenase; *GPT*: glutamate-pyruvate transaminase; *OGDH*: 2-oxoglutarate/α-ketoglutarate dehydrogenase; *OGDHL*: 2-oxoglutarate/α-ketoglutarate dehydrogenase dehydrogenase like; *SLC25A22*: solute carrier family 25 member 22 = mt-glutamate carrier). Data are presented as box plots indicating median, 25th–75th percentile (box) and median±1.5·IQR (whiskers), minimum and maximum values (dots). Differences in mean values were tested for significance using multiple t-test followed by Benjamini-Hochberg correction for multiple testing. **e** Detailed annotation of single up-regulated TCA-cycle key-enzymes in the severe (orange) or mild (blue) respiratory phenotype samples. Blue boxes mark steps mainly involved in glutamate- and α-ketoglutarate-linked electron transfer and orange boxes highlight steps related to fatty acid, pyruvate and succinate mediated electron transfer. Substrates used in HRR analysis (malate, glutamate, pyruvate, and succinate) are indicated in bold type while orange and blue colored names and arrows mark enzymes and enzymatic reactions that are upregulated in samples exhibiting either a severe or mild respiratory phenotype, respectively.

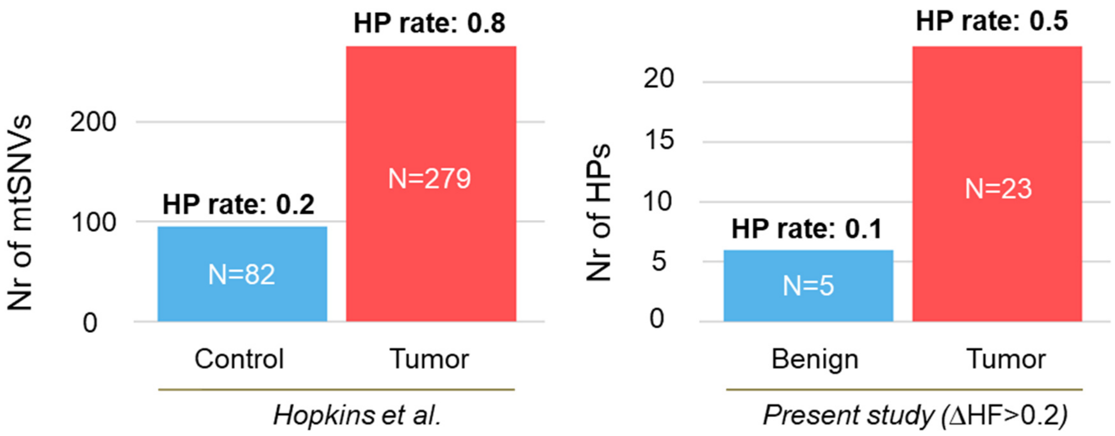

Supplementary Figure 8 Comparison of mutation numbers and rates. Our list of mitochondrial heteroplasmy (HP) variants identified in 50 paired benign tissue samples was re-assessed using the threshold for mutation calling applied in the study of Hopkins et al.[8] (Difference in allele frequency >0.2) and numbers and rates were compared. Note that control samples were blood samples in the study of Hopkins et al. and benign prostate tissue samples in the present study.

Supplementary References

1. Gnaiger E, *et al.* Mitochondrial respiratory states and rates. *Preprint at MitoFit Preprint Archive* https://www.mitofit.org/images/4/46/Gnaiger_2019_MitoFit_Preprint_Arch_doi_10.26124_mitofit_190001.pdf (2019). Doi:doi:10.26124/mitofit:190001.v6.

2. Gnaiger E, Kuznetsov AV. Mitochondrial respiration at low levels of oxygen and cytochrome c. *neu* **30**, 252-258 (2002). Doi:10.1042/.

3. Gnaiger E. Capacity of oxidative phosphorylation in human skeletal muscle: new perspectives of mitochondrial physiology. *Int J Biochem Cell Biol* **41**, 1837-1845 (2009). Doi:10.1016/j.biocel.2009.03.013.

4. Lindberg J, *et al.* The mitochondrial and autosomal mutation landscapes of prostate cancer. *Eur Urol* **63**, 702-708 (2013). Doi:10.1016/j.eururo.2012.11.053.

5. Ju YS, *et al.* Origins and functional consequences of somatic mitochondrial DNA mutations in human cancer. *Elife* **3**, 02935 (2014). Doi:10.7554/eLife.02935.

6. McCrow JP, *et al.* Spectrum of mitochondrial genomic variation and associated clinical presentation of prostate cancer in South African men. *Prostate* **76**, 349-358 (2016). Doi:10.1002/pros.23126.

7. Kalsbeek AM, *et al.* Mutational load of the mitochondrial genome predicts pathological features and biochemical recurrence in prostate cancer. *Aging (Albany NY)* **8**, 2702-2712 (2016). Doi:10.18632/aging.101044.

8. Hopkins JF, *et al.* Mitochondrial mutations drive prostate cancer aggression. *Nat Commun* **8**, 656 (2017). Doi:10.1038/s41467-017-00377-y.
