# Supplementary material for: OXPHOS remodeling in high-grade prostate cancer involves mtDNA mutations and increased succinate oxidation
Source: Nat Commun. 2020 Mar 20;11:1487. doi: 10.1038/s41467-020-15237-5 (PMC7083862; doi:10.1038/s41467-020-15237-5)
Supplement: Supplementary file 3 — Description of Additional Supplementary Files [file 41467_2020_15237_MOESM3_ESM.pdf]

Description of Additional Supplementary Files

File Name: Supplementary Data 1
Description: **mtDNA heteroplasmies**. Heteroplasmy variants exhibiting a heteroplasmy level >2%. Locations, heteroplasmy variants, gene location, heteroplasmy level, report status (0=not reported and 1=previously reported) and amino acid substitution caused by the heteroplasmies found in either both tissues (shared heteroplasmies), only the benign (private benign heteroplasmies) or only the malignant (private cancer heteroplasmies) tissues.
