# Supplementary material for: OXPHOS remodeling in high-grade prostate cancer involves mtDNA mutations and increased succinate oxidation
Source: Nat Commun. 2020 Mar 20;11:1487. doi: 10.1038/s41467-020-15237-5 (PMC7083862; doi:10.1038/s41467-020-15237-5)
Supplement: Supplementary file 5 — Reporting Summary [file 41467_2020_15237_MOESM5_ESM.pdf]

nature research

Corresponding author(s): Helmut Klocker

Last updated by author(s): Feb 20, 2020

# Reporting Summary

Nature Research wishes to improve the reproducibility of the work that we publish. This form provides structure for consistency and transparency in reporting. For further information on Nature Research policies, see Authors & Referees and the Editorial Policy Checklist .

## Statistics

For all statistical analyses, confirm that the following items are present in the figure legend, table legend, main text, or Methods section.

| n/a | Confirmed | |
|---|---|---|
| ☐ | ☒ | The exact sample size ($n$) for each experimental group/condition, given as a discrete number and unit of measurement |
| ☐ | ☒ | A statement on whether measurements were taken from distinct samples or whether the same sample was measured repeatedly |
| ☐ | ☒ | The statistical test(s) used AND whether they are one- or two-sided<br>*Only common tests should be described solely by name; describe more complex techniques in the Methods section.* |
| ☐ | ☒ | A description of all covariates tested |
| ☐ | ☒ | A description of any assumptions or corrections, such as tests of normality and adjustment for multiple comparisons |
| ☐ | ☒ | A full description of the statistical parameters including central tendency (e.g. means) or other basic estimates (e.g. regression coefficient) AND variation (e.g. standard deviation) or associated estimates of uncertainty (e.g. confidence intervals) |
| ☐ | ☒ | For null hypothesis testing, the test statistic (e.g. $F$, $t$, $r$) with confidence intervals, effect sizes, degrees of freedom and $P$ value noted<br>*Give P values as exact values whenever suitable.* |
| ☒ | ☐ | For Bayesian analysis, information on the choice of priors and Markov chain Monte Carlo settings |
| ☒ | ☐ | For hierarchical and complex designs, identification of the appropriate level for tests and full reporting of outcomes |
| ☐ | ☒ | Estimates of effect sizes (e.g. Cohen's $d$, Pearson's $r$), indicating how they were calculated |

*Our web collection on statistics for biologists contains articles on many of the points above.*

## Software and code

Policy information about availability of computer code

| | |
|---|---|
| Data collection | For data collection we used Excel (V2016 and V2000, Microsoft, Redmont, WA), DatLab Software V6 and 7 (Oroboros Instruments, Innsbruck Austria), PROSize (Advanced Analytical Technologies, Ames, IA), Ion Torrent Suit (Life Technologies, Waltham, MA) and Illumina HiSeq 2500 Instrument Software (Illumina, San Diego, CA). |
| Data analysis | Data analyses were performed utilizing commercially or publicly available software. Software tools, including versions, references and links are provided in the Methods section. |

For manuscripts utilizing custom algorithms or software that are central to the research but not yet described in published literature, software must be made available to editors/reviewers. We strongly encourage code deposition in a community repository (e.g. GitHub). See the Nature Research guidelines for submitting code & software for further information.

## Data

Policy information about availability of data

All manuscripts must include a data availability statement. This statement should provide the following information, where applicable:
- Accession codes, unique identifiers, or web links for publicly available datasets
- A list of figures that have associated raw data
- A description of any restrictions on data availability

Data that support the findings of this study are available within the article and its supplementary files or from the authors upon reasonable request. Supplementary Information provides Supplementary Tables and Figures, Supplementary Data 1 provides a list of mtDNA Heteroplasmies, the Source Data File provides data sets underlying Fig. 2-8 and Supplementary Fig. 1-4 and 6-8. The Supplementary Software iSee Package for 3D visualization of Complex I mutations is provided for download or online access at [https://github.com/genepi/mt-c1]. DNA and RNA sequence data sets have been disposed in EGA (European Genome-Phenome Archive [www.ega-archive.org]) under the following accession numbers: EGAD00001005931 (RNAseq data set, [https://ega-archive.org/datasets// EGAD00001005931] and EGAD00001005945 (mtDNA data set, [https://ega-archive.org/datasets//EGAD00001005945]. Prostate cancer gene expression datasets with survival information were accessed at the Cancer Genome Atlas (TCGA) Data Portal ([https://cancergenome.nih.gov/]): TCGA-PRAD ([https:// portal.gdc.cancer.gov/projects/TCGA-PRAD]) and the Gene Expression Omnibus portal ([https://www.ncbi.nlm.nih.gov/geo/]): GSE16560 ([https://

www.ncbi.nlm.nih.gov/geo/query/acc.cgi?acc=GSE16560]), GSE40272 ([https://www.ncbi.nlm.nih.gov/geo/query/acc.cgi?acc=GSE40272]), GSE70768 ([https://www.ncbi.nlm.nih.gov/geo/query/acc.cgi?acc=GSE70768]) and GSE70769 ([https://www.ncbi.nlm.nih.gov/geo/query/acc.cgi?acc=GSE70769]). Protein structure data were accessed at the Protein Data Base (PDB, [https://www.rcsb.org/]: 4HE8 ([https://www.rcsb.org/structure/4HE8]), 4HEA ([https://www.rcsb.org/structure/4HEA]), 5STD ([https://www.rcsb.org/structure/5XTD]), 5XTC ([https://www.rcsb.org/structure/5XTC]).

# Field-specific reporting

Please select the one below that is the best fit for your research. If you are not sure, read the appropriate sections before making your selection.

[x] Life sciences  [ ] Behavioural & social sciences  [ ] Ecological, evolutionary & environmental sciences

For a reference copy of the document with all sections, see nature.com/documents/nr-reporting-summary-flat.pdf

# Life sciences study design

All studies must disclose on these points even when the disclosure is negative.

| | |
|---|---|
| Sample size | 50 paired malignant/nonmalignant tissue samples |
| Data exclusions | no data were excluded |
| Replication | Measurement of split tissue samples in HRR analysis, 3 NGS runs for mtDNA samples, paired-end NGS for RNA samples, 3-6 biological replicates for cell line HRR analysis. |
| Randomization | No randomization. Groups according to clinical parameters (tumor grading, patients age, PSA and free serum levels, respirometry pattern, presence/absense of mutations, gene expression signature) |
| Blinding | No blinding |

# Reporting for specific materials, systems and methods

We require information from authors about some types of materials, experimental systems and methods used in many studies. Here, indicate whether each material, system or method listed is relevant to your study. If you are not sure if a list item applies to your research, read the appropriate section before selecting a response.

## Materials & experimental systems

| n/a | Involved in the study |
|---|---|
| [ ] | [x] Antibodies |
| [ ] | [x] Eukaryotic cell lines |
| [x] | [ ] Palaeontology |
| [x] | [ ] Animals and other organisms |
| [ ] | [x] Human research participants |
| [ ] | [x] Clinical data |

## Methods

| n/a | Involved in the study |
|---|---|
| [x] | [ ] ChIP-seq |
| [x] | [ ] Flow cytometry |
| [x] | [ ] MRI-based neuroimaging |

## Antibodies

| | |
|---|---|
| Antibodies used | anti-p63 (4A4) Mouse Monoclonal Primary Antibody, Roche, #05867061001<br>Monoclonal Rabbit Anti-Human P504S (AMACR) Clone 13H4, #M3616, DAKO-Agilent<br>anti-VADC1 (D73D12, Cell Signaling Technologies, Leiden, The Netherlands; 1:400,)<br>anti-SDHA (D6J9M, Cell Signaling Technologies; 1:400)<br>anti-NDUFS4 (EP7832, Abcam, Cambridge, UK; 1:200) |
| Validation | IHC antibodies used in routine and/or validated by provider. Antibodies used in previouse studies (doi: 10.1038/pcan.2013.4, 10.1155/2018/1347174) |

## Eukaryotic cell lines

Policy information about cell lines

| | |
|---|---|
| Cell line source(s) | LNCaP and PC3 prostate cancer and benign prostate epithelial RWPE1 cell lines were purchased from the American Type Culture Collection (ATCC; Rockville, MD). DuCaP prostate cancer cells were obtained from Radboud University, Nijmegen, The Netherlands. The human telomerase reverse transcriptase-immortalized benign prostate epithelial cell line EP156T was established by hTERT overexpression (doi: 10.1158/0008-5472.CAN-05-2183). |

| | |
|---|---|
| Authentication | Short tandem repeat analysis using the AmpFlSTR® SGM Plus® PCR amplification kit (Applied Biosystems, Waltham, MA) |
| Mycoplasma contamination | Regular testing, Venor GeM-qEP PCR based assay, BioProducts, #11-9025 |
| Commonly misidentified lines (See ICLAC register) | Not included |

## Human research participants

Policy information about studies involving human research participants

| | |
|---|---|
| Population characteristics | Prostate cancer patients undergoing radical prostatectomy as a first line treatment. Patients received standard of care radical prostatectomies for treatment of porstate cancer. |
| Recruitment | University Hospital for Urology, Medical University of Innsbruck |
| Ethics oversight | Ethics Committee of the Medical University Innsbruck |

Note that full information on the approval of the study protocol must also be provided in the manuscript.

## Clinical data

Policy information about clinical studies

All manuscripts should comply with the ICMJEguidelines for publication of clinical research and a completedCONSORT checklist must be included with all submissions.

| | |
|---|---|
| Clinical trial registration | Study with tissue samples of patients who received standard of care radical prostatectomy, no clinical trial |
| Study protocol | Patients underwent standard-of-care radical prostatectomy. Tissue samples for the study were afterwards excised from the radical prostectomy specimens by a certified uropathologist. |
| Data collection | Tissue Archive Database of Prostate Cancer Biobank (Departments of Urology and Pathology, Medical University Innsbruck) |
| Outcomes | PSA serum level, tumor histopathology |
